# Unusual Presentations of Intramuscular Lipoma: A Case Series

**DOI:** 10.7759/cureus.73654

**Published:** 2024-11-13

**Authors:** Jenan Hamad, Zainab AlQubaiti, Fatema Hubail, Huda AlSahlawi

**Affiliations:** 1 General Practice, Salmaniya Medical Complex, Manama, BHR; 2 Radiology, Salmaniya Medical Complex, Manama, BHR

**Keywords:** benign tumors, case series, intramuscular lipoma, lipoma, magnetic resonance imaging, muscle tissue, soft tissue tumors, surgical excision, ultrasound

## Abstract

Intramuscular lipomas (IMLs) are rare benign tumors that arise when lipomas infiltrate muscle tissue. These tumors are most commonly found in the large muscles of the limbs and trunk, particularly in the thigh, shoulder, and upper arm. IMLs often present as painless masses but can cause pain, muscle dysfunction, and neurological deficits as they grow and invade surrounding structures. This case series discusses three patients with IMLs located in uncommon sites: the wrist, foot, and trapezius muscle. Diagnostic imaging, including ultrasound and magnetic resonance imaging (MRI), was essential in identifying and characterizing these lesions. Surgical excision is the treatment of choice, though the infiltrative nature of IMLs increases the risk of recurrence. This series underscores the importance of early diagnosis and appropriate management of IMLs, particularly in rare anatomical locations.

## Introduction

Lipomas are the most common benign soft tissue tumors of mesenchymal origin. They are categorized into two main types: superficial lipomas, which involve mature fatty tissue located in the subcutaneous layer, and deep lipomas, found beneath the dermis and within the investing fascia [1,2]. A rarer subtype, the intramuscular lipoma (IML), arises when a lipoma infiltrates muscle layers, accounting for less than 1% of all lipomas [3,4]. IMLs are typically found in the large muscles of the limbs and trunk, especially in the thigh, shoulder, and upper arm, but they are much rarer in areas like the hand and foot. Although IMLs are relatively common in the shoulder region, they are particularly rare in the trapezius muscle, with only a few cases reported [5]. In this case series, we present three cases of IMLs found in uncommon locations: the wrist, foot, and trapezius muscles of the shoulder. We discuss their clinical features and imaging characteristics, highlighting the rarity of these atypical sites of occurrence.

## Case presentation

Case 1

A 62-year-old female with a history of hypothyroidism was referred from the local healthcare center to the general surgery outpatient clinic due to a one-year history of swelling on the dorsal surface of her left wrist. The patient reported gradual enlargement of the swelling, accompanied by intermittent pain, alleviated by Voltaren. She also experienced tingling and weakness in the left wrist, which occurred only during episodes of pain. She denied any history of numbness or restricted joint movement.

On clinical examination, a solid, soft, and tender mass measuring approximately 1.5 x 2 x 2.5 cm was palpated on the dorsal aspect of the ulnar side of the left wrist. The patient demonstrated a full range of motion in the left wrist without pain and maintained good grip strength in the left hand.

**Figure 1:**
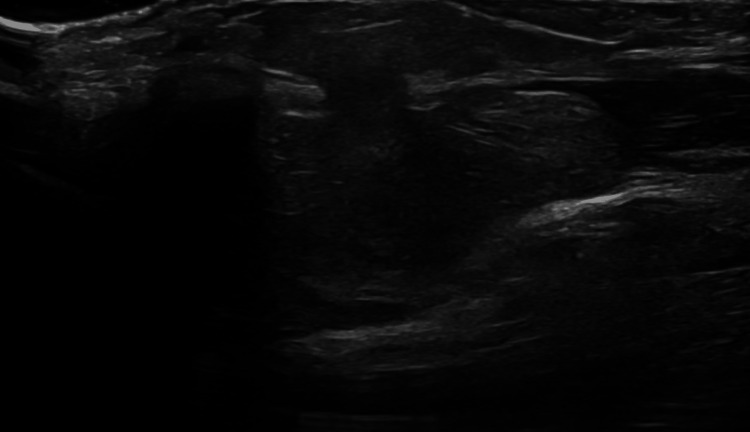
Ultrasound image of the left wrist showing a subcutaneous lobulated soft tissue mass on the dorsal ulnar side, measuring 1.5 cm x 2 cm x 2.5 cm. The lesion is well-encapsulated with homogeneous echotexture. A superficial component is radially located in relation to the intact extensor carpi ulnaris tendon, while a deep component extends toward the underlying distal radioulnar joint space.

Ultrasound imaging of the left wrist revealed a well-defined, encapsulated, lobulated mass in the subcutaneous tissue overlying the dorsal aspect of the ulnar side of the wrist. The mass had a superficial component located radial to the extensor carpi ulnaris tendon, which appeared intact, and a deeper component that seemed to connect to the underlying distal radioulnar joint space. No significant internal vascularity or calcification was observed. These findings suggested the possibility of an intra-articular lipoma, likely arising from the dorsal aspect of the distal radioulnar joint (Figure 1). Follow-up ultrasound imaging conducted after one year showed no changes in the size or characteristics of the mass.

**Figure 2:**
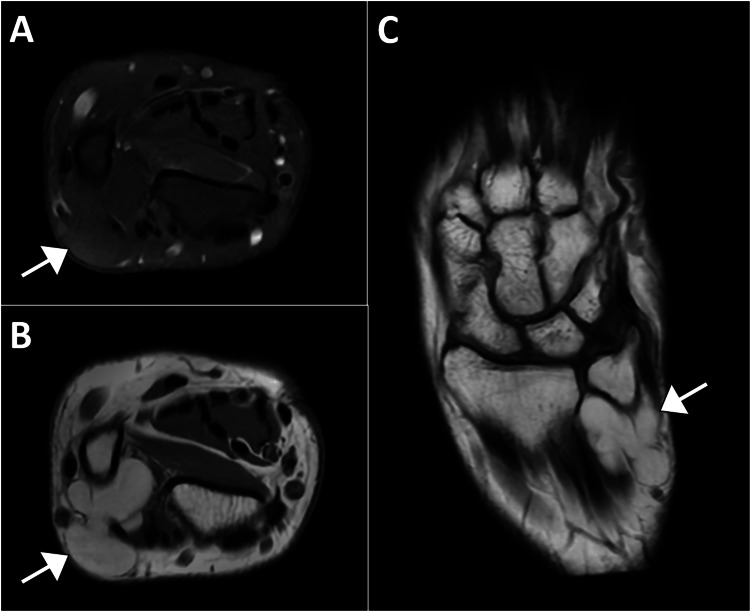
Magnetic resonance imaging of the left wrist (Panel A displays an axial proton density short tau inversion recovery image, Panel B shows an axial T1-weighted image without fat saturation, and Panel C shows a coronal proton density image) showing a fat-containing mass (arrow) within the extensor indices muscle. The mass adheres to the posterior wall of the tendon, pierces the muscle fascia, and extends into the subcutaneous tissue, giving it a bilobed appearance. It measures 2.2 cm x 2 cm and extends 2.2 cm along the longitudinal axis. The lesion is near the neurovascular bundle and abuts the radio-ulnar interosseous membrane, without intra-articular extension.

Magnetic resonance imaging performed later showed fat signal intensity across all sequences, with homogeneous suppression on fat-suppressed sequences. The mass appeared to originate from the extensor indices muscle, where it adhered to the posterior wall of the tendon, pierced the muscle fascia, and extended superficially into the subcutaneous tissue, giving a bilobed shape. The lesion measured 2.2 cm in anterior-posterior diameter and 2 cm in transverse diameter, extending 2.2 cm along the longitudinal axis. The lesion abutted the radio-ulnar interosseous membrane and was in close proximity to the neurovascular bundle, positioned on the radial side in relation to the extensor carpi ulnaris tendon. It abutted the distal radioulnar joint without definite intra-articular extension. These findings were consistent with an intramuscular lipoma in the extensor indices muscles, extending into the overlying subcutaneous tissue (Figure 2). Given the patient's symptoms and imaging findings, surgical excision of the lipoma was recommended. The patient was given time to consider her treatment options.

Case 2

A 47-year-old woman with a three-year history of hypothyroidism and diabetes mellitus was referred to the vascular surgery outpatient clinic from the local healthcare center for evaluation of a left dorsal foot swelling, associated with localized discomfort. The patient reported a palpable lump on the dorsal aspect of the left foot. The mass had remained stable in size with no signs of trauma, skin discoloration, or itching, and it did not affect her mobility. A diagnostic ultrasound was ordered to investigate the nature of the mass.

**Figure 3:**
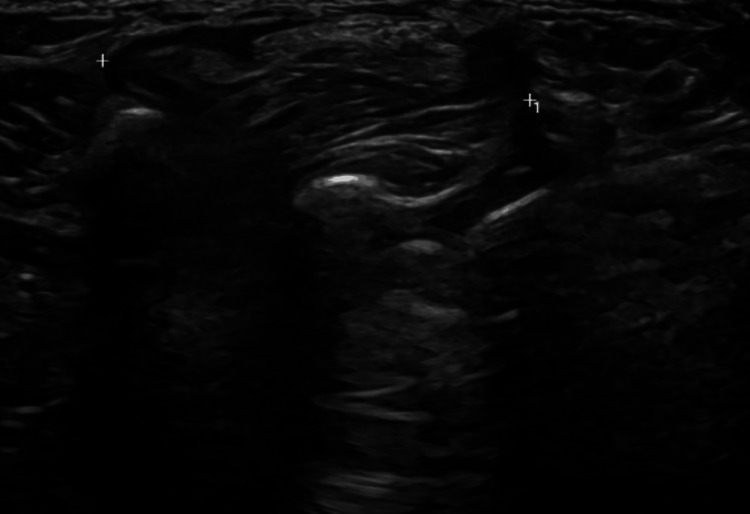
Ultrasound image of the left dorsal foot region showing a 2.8 cm x 4.8 cm x 1.1 cm soft tissue mass, located superficially to the tarsal bones with posterior extension toward the intertarsal joints. The mass is well-encapsulated, exhibiting altered echotexture with multiple echogenic stripes, but no internal vascularity, calcification, or cystic features.

The ultrasound revealed a well-defined soft tissue mass located superficially over the tarsal bones, measuring 2.8 x 4.8 x 1.1 cm. The mass was encapsulated, with a clear boundary at the superficial aspect, while the posterior portion extended into the underlying structures, suggesting an origin from the intertarsal joints. No internal vascularity was observed, and the mass showed an altered echotexture with multiple echogenic stripes. There was no focal calcification or cystic components. Based on these characteristics, the mass was likely diagnosed as a lipoma with a possible origin from the intertarsal joint (Figure 3). Magnetic resonance imaging was recommended for further evaluation.

**Figure 4:**
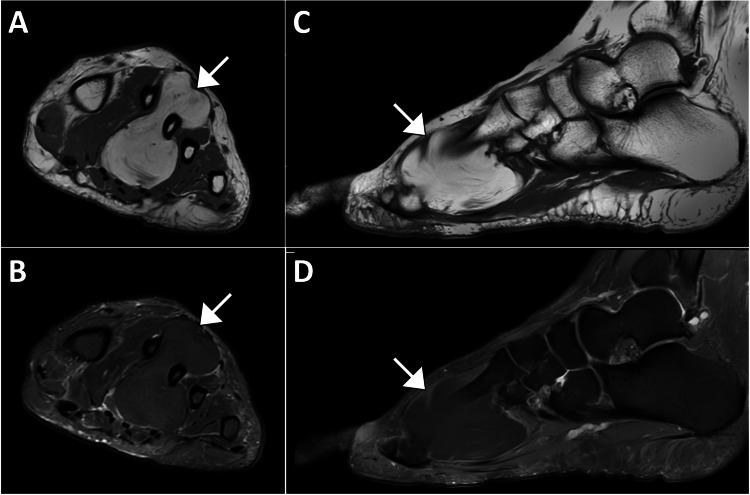
Magnetic resonance imaging of the left foot (Panel A shows a coronal T1-weighted image without fat saturation, Panel B shows a coronal T1-weighted image with fat saturation, Panel C shows a sagittal T1-weighted image without fat saturation, and Panel D shows a sagittal T1-weighted image with fat saturation) showing a large bilobed intramuscular lesion with both plantar and dorsal components, measuring 4.3 cm in anterior-posterior diameter, 2.5 cm transversely, and 4.5 cm longitudinally. The lesion demonstrates fat signal intensity on all sequences, with complete suppression on fat-saturated images. Likely originating from the dorsal interosseous muscle, the lesion decompresses through the second webspace to the plantar side, with potential involvement of the adductor hallucis longus muscle.

After reviewing the ultrasound findings, the vascular surgery team cleared the patient as there were no vascular concerns. She was subsequently referred to orthopedic surgery for further management. Magnetic resonance imaging confirmed the presence of a large lobulated lesion with intramuscular fat signal intensity, consistent with a lipoma. The lesion appeared to originate from the dorsal interosseous muscle and extended through the second webspace, reaching the plantar side of the foot. There was also potential involvement of the adductor hallucis longus muscle. Given the location and possible involvement of adjacent muscles, orthopedic follow-up was recommended (Figure 4). The patient was offered the option of surgical excision or conservative management. Considering her history of diabetes and associated risks, she opted for conservative management.

Case 3

A 36-year-old male with no prior medical history was referred from the local healthcare center to the general surgery department in 2020 due to a four-year history of left neck swelling. The swelling had previously been diagnosed as a lipoma, and he was advised to undergo excision under local anesthesia; however, he did not follow up for the procedure. The swelling, initially tennis-ball-sized, progressively increased over the years, prompting him to seek medical advice in 2020. He reported no pain, paraesthesia, or restricted shoulder movement, and denied changes in voice, shortness of breath, difficulty swallowing, or weight loss.

**Figure 5:**
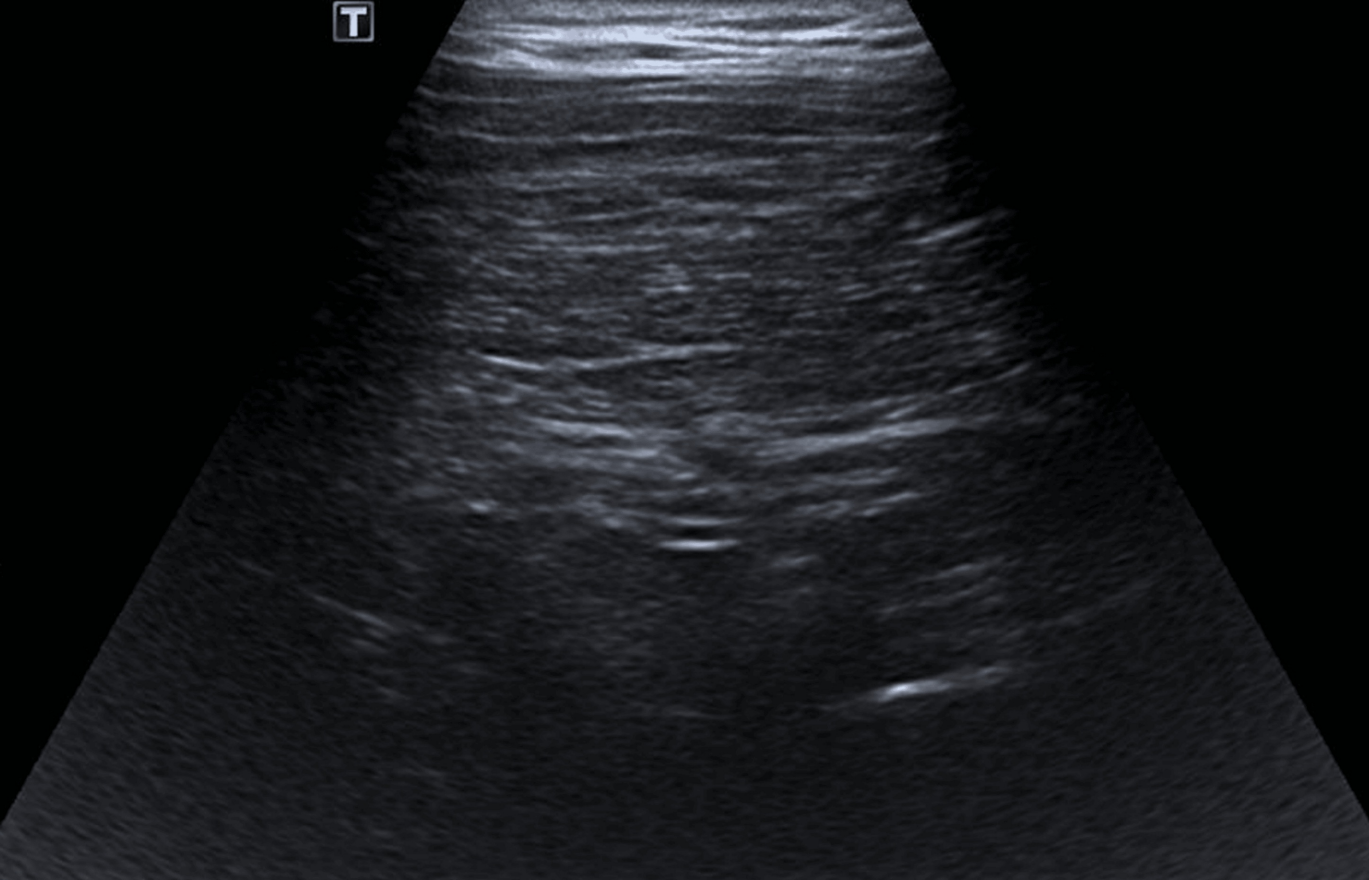
Ultrasound image of the left neck and upper back showing a large, encapsulated, iso- to mildly hyperechoic mass measuring over 11 cm. The mass is located approximately 1.1 cm from the skin, superficial to the left subclavian vein. It shows no posterior acoustic shadowing, internal vascularity, calcifications, or nodular soft tissue components.

Initial ultrasound revealed a large lipoma on the left lateral neck, extending to the upper back. The mass was hyperechoic, measuring over 11 cm, with poorly visualized margins. It was situated about 1.1 cm from the skin and lay superficial to the left subclavian vein, with no posterior acoustic shadowing, calcifications, or internal vascularity. Sub-centimeter cervical lymph nodes with preserved fatty hilum were noted bilaterally, with no significant lymphadenopathy. The thyroid gland appeared normal. The ultrasound findings were suggestive of a lipoma (Figure 5).

The patient was advised to undergo a computed tomography scan to assess the mass’s margins, its relationship to surrounding vascular structures, and to consider a fine needle aspiration biopsy; however, he did not follow up.

In 2023, the patient presented to the Accident & Emergency department due to an increase in the size of the mass, though he remained asymptomatic. Examination revealed a rubbery, non-tender swelling extending from the supraclavicular area to the left side of the neck, measuring 15 x 5 cm, not attached to the skin or muscle.

**Figure 6:**
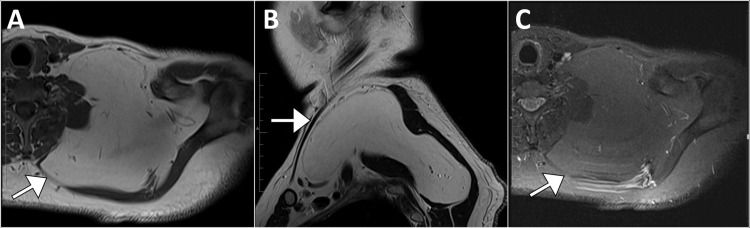
Magnetic resonance imaging (Panel A shows an axial T1-weighted image without fat saturation, Panel B shows a sagittal T1-weighted image without fat saturation, and Panel C shows an axial T1-weighted image with fat saturation) showing a large bilobed intramuscular mass within the left trapezius muscle, following fat signal intensity on all sequences with complete suppression on short tau inversion recovery images. The lesion measures 13.5 cm in anterior-posterior diameter, 16.5 cm transversely, and 13.1 cm longitudinally. Despite its size, the lesion shows no significant post-contrast enhancement or concerning features. It extends into the subacromial fat and around the supraspinatus muscle but does not infiltrate the rotator cuff. The lesion abuts the posterior aspect of the acromioclavicular joint without intra-articular involvement, and is inseparable from the cervical neurovascular bundle, with vessels from the subclavian artery traversing the mass.

Magnetic resonance imaging of the left shoulder revealed a large intramuscular mass within the left trapezius muscle, showing fat signal intensity across all sequences with complete suppression in STIR images. No significant post-contrast enhancement or concerning features were noted despite the mass’s large size. The lesion had a bilobed configuration, measuring 13.1 cm in anteroposterior diameter, 16.5 cm in transverse diameter, and 13.1 cm longitudinally. The mass extended into the subacromial fat and around the supraspinatus muscle, without clear involvement of the rotator cuff muscles. It also extended to abut the posterior aspect of the acromioclavicular joint, with no definite intra-articular extension. The lesion appeared inseparable from the cervical neurovascular bundle at the thyroid level, with traversing vessels arising from the subclavian artery (Figure 6). The patient is currently under follow-up with the general surgery team and is scheduled for lipoma resection for cosmetic reasons only.

## Discussion

Lipomas are the most common benign mesenchymal tumors, primarily composed of mature adipose tissue, and can develop in any fat-containing area of the body [3]. Classified by the World Health Organization, lipomas encompass subtypes such as lipoma, lipomatosis, and angiolipoma, with further divisions into superficial and deep subtypes [1]. Superficial lipomas are confined to the subcutaneous tissue, the most frequent site for these growths, while deeper lipomas occur beneath the dermis or deep fascia. These deeper lesions can appear in intermuscular spaces, affecting connective tissue, or within muscle fibers where they may impact muscle function [1].

IMLs, a rare subtype, account for less than 1% of all lipomas. Although lipomas in general comprise approximately 16% of all soft tissue mesenchymal tumors [6], IMLs primarily affect adults aged 40 to 70, with a slight male predominance [3,7]. While the pathogenesis and etiology of IMLs are not fully understood, IMLs, like other lipomas, are believed to arise from neoplastic processes originating in multipotent mesenchymal cells, although a reactive pathogenesis has also been proposed. Various factors, including trauma, chronic irritation, obesity, developmental disorders, endocrine or metabolic conditions, and genetic predisposition, have been suggested to contribute to IML development [3].

There is some evidence to support a link between body mass index and the formation of IMLs. Ramos-Pascua et al. observed that two-thirds of cases in their study involved overweight or obese individuals, although no familial cases have been documented to date [3]. Additional theories propose that neurogenic or myogenic factors could influence IML formation. Mori et al. found that IMLs, particularly the infiltrative subtype, often show selective muscle fiber atrophy or degeneration, possibly associated with elevated levels of high mobility group proteins, which may facilitate the infiltrative growth of these tumors. Interestingly, this muscle fiber atrophy is not limited to areas of fatty infiltration but may also be observed in surrounding muscle fibers [3,6].

IMLs predominantly arise within large muscles of the extremities, particularly the thigh, shoulder, and upper arm [1], with the quadriceps femoris being the most frequently involved muscle, followed by the deltoid [8]. Although IMLs can develop in various locations, they are exceedingly rare in the hand and forearm, representing less than 1% of all lipomas. There have been occasional reports of IMLs in more unusual locations, such as the thenar muscle and the thumb, though these cases are infrequent [6]. In our case series, we observed IMLs in the left wrist (extensor indices muscle), left foot (dorsal interosseous muscle), and left trapezius muscle-locations that have been rarely documented in the literature and for which limited information is available.

The clinical presentation of IMLs varies depending on the tumor's size and location [6]. They are most often identified as painless swellings with minimal or no symptoms in the early stages. However, pain may occur in larger or deeper lipomas, typically due to compression of surrounding tissues or nerves [1,3]. In some cases, patients may experience paresthesia or neurological deficits from nerve compression [4]. Muscle dysfunction or cramping may also occur if the lipoma infiltrates muscle tissue [6]. In advanced stages, patients may encounter reduced range of motion or functional limitations due to mechanical restrictions [3]. Unusual presentations of wrist IMLs, such as trigger finger and carpal tunnel syndrome, have also been reported [9]. In the shoulder region, deep lipomas may cause brachialgia, mimicking thoracic outlet syndrome, impingement syndrome, or suprascapular nerve entrapment symptoms [10]. These symptoms can persist for extended periods, often ranging from months to years, and may sometimes lead to misdiagnosis if mistaken for more common conditions affecting the area [3,6].

Imaging plays a crucial role in diagnosing IMLs, with ultrasound being a common initial modality due to its convenience, efficiency, and cost-effectiveness [6]. On ultrasound, a well-circumscribed IML is typically characterized by a hyperechoic ovoid mass with defined borders separating it from surrounding muscle, resembling a superficial lipoma with no blood flow signals. Areas of heterogeneity may also be present, indicating thin intrinsic septa [1,6]. However, encapsulated IMLs may be difficult to visualize, as ultrasound may not clearly show their bilobed structure or intramuscular extensions [1,2]. In infiltrative variants, the tumor’s fatty content separates muscle tissue, creating a heterogeneous striated appearance that could be misinterpreted as a non-lipomatous lesion, potentially raising concerns for malignancy [1].

To overcome limitations of ultrasound and obtain a definitive diagnosis, computed tomography scans and magnetic resonance imaging are often employed. These imaging modalities are crucial in evaluating the anatomical relationships of IMLs with adjacent structures [1,3]. Computed tomography scans are effective for identifying lipomas, typically revealing IMLs as hypodense masses with negative Hounsfield values similar to adipose tissue, often displaying soft tissue streaks within the lesion that are more clearly defined on computed tomography than on magnetic resonance imaging. Computed tomography also provides clearer detection of any ossification [3].

Magnetic resonance imaging, however, is the preferred modality for IML assessment due to its superior ability to confirm the diagnosis and identify atypical features indicative of other pathologies [11]. Magnetic resonance imaging is particularly effective in differentiating fat-containing tumors from other soft tissue masses and in distinguishing between various types of lipomatous lesions [4].

IMLs exhibit diverse morphological features, appearing as round, ovoid, or fusiform masses on magnetic resonance imaging with either homogeneous or heterogeneous signal intensities [4]. They typically present as homogeneous fatty masses with high signal intensity on both T1- and T2-weighted images, consistent with typical lipomas. However, some IMLs show inhomogeneity, with regions of reduced signal intensity that indicate muscle or fibrous tissue presence. Magnetic resonance imaging also helps differentiate IMLs from well-differentiated liposarcomas, which may appear similarly due to their lipomatous composition. Features such as uninodularity, well-defined margins, and muscle fibers within the lesion generally suggest a benign etiology [1], while liposarcomas tend to be larger, multilobular masses with nodules comprising heterogeneous tissue [3]. Magnetic resonance imaging's detailed imaging is essential for preoperative planning by providing crucial information on tumor size, shape, and anatomical relationships that surpass the detail provided by computed tomography [2].

Despite the value of imaging, biopsy remains an essential diagnostic tool, especially for larger or complex lipomas, to exclude the possibility of liposarcoma. Guided by magnetic resonance imaging findings, biopsies are critical in establishing a definitive diagnosis. In cases where differentiation from malignancies like liposarcoma is necessary, excisional biopsy or fine-needle aspiration cytology may be required [1]. Histopathological analysis is necessary for a comprehensive diagnosis and provides essential guidance for treatment decisions.

The treatment of IMLs is guided by the size, location, and associated symptoms of the mass [3]. Conservative management, involving observation and reassurance, may be considered for small, asymptomatic lesions but is unsuitable for symptomatic lipomas [2,3]. Surgical excision is the treatment of choice, especially for masses larger than 5 cm or those causing functional impairment, muscle dysfunction, neurological symptoms, or requested for cosmetic reasons. Marginal excision is generally preferred, though wider excision with clear margins is advised if malignancy is suspected on magnetic resonance imaging or biopsy to reduce recurrence risk. In cases where complete excision is not feasible, debulking may be an alternative [2,3].

The recurrence rate for IMLs is high, ranging from 50% to 80%, largely due to their infiltrative nature. Studies indicate that recurrence rates can be significantly lowered with wide excision; for instance, a clinical study from Korea found that the recurrence rate was 11.1% following marginal excision, whereas wide excision reduced recurrence to 0% [12]. Follow-up is recommended when atypical cellular features are observed post-excision [2].

## Conclusions

IMLs are rare benign tumors that can cause significant clinical challenges due to their infiltrative nature and location within muscle tissue. Although IMLs are typically asymptomatic, they can lead to pain, muscle dysfunction, neurological deficits, and functional impairments as they grow. Diagnostic imaging, particularly magnetic resonance imaging, plays a crucial role in identifying IMLs and differentiating them from other soft tissue masses, with histopathological analysis remaining essential for definitive diagnosis. Surgical excision remains the treatment of choice, especially for symptomatic or large lesions, though the infiltrative nature of IMLs increases the risk of recurrence. Early diagnosis and appropriate surgical intervention are critical in managing these lesions and minimizing functional impairment, with careful follow-up needed to monitor for recurrence.

## References

[1] Papakostas T, Tsovilis AE, Pakos EE (2016). Intramuscular lipoma of the thenar: a rare case. Arch Bone Jt Surg.

[2] Soni A, Mehta M, Shirodkar K, Singh A, Talawadekar G (2023). A case of intramuscular lipoma of thenar eminence with a short review of published hand lipoma case reports. Cureus.

[3] McTighe S, Chernev I (2014). Intramuscular lipoma: a review of the literature. Orthop Rev (Pavia).

[4] Byeon JY, Hwang YS, Lee JH, Choi HJ (2023). Recurrent intramuscular lipoma at extensor pollicis brevis: a case report. World J Clin Cases.

[5] Meza-Hernandez J, Gonzalez-Cantú I, Del Carmen-Ortega I, Vázquez-Sánchez HA, Apellaniz-Campo AG (2024). Giant lipoma in the trapezius muscle: a rare case report. Aesthet Surg J Open Forum.

[6] Tian H, Qu WR, Pan J, Zhu Z, Liu J, Li R (2020). Lipoma in the pronator quadratus: a case report. Medicine (Baltimore).

[7] Ramos-Pascua LR, Guerra-Álvarez OA, Sánchez-Herráez S, Izquierdo-García FM, Maderuelo-Fernández JÁ (2013). [Intramuscular lipomas: large and deep benign lumps not to underestimated. Review of a series of 51 cases]. Rev Esp Cir Ortop Traumatol.

[8] Lui TH (2013). Intramuscular lipoma of the abductor digiti minimi mimicking intramuscular haemangioma. BMJ Case Rep.

[9] Huang C, Jin HJ, Song DB (2021). Trigger finger at the wrist caused by an intramuscular lipoma within the carpal tunnel: a case report. World J Clin Cases.

[10] Elbardouni A, Kharmaz M, Salah Berrada M, Mahfoud M, Elyaacoubi M (2011). Well-circumscribed deep-seated lipomas of the upper extremity. A report of 13 cases. Orthop Traumatol Surg Res.

[11] Rispoli L, Singh JR, Piesco J (2017). Unusual location of intramuscular lipoma presenting as an extensor tendon tear: a diagnostic dilemma. Am J Phys Med Rehabil.

[12] Han HH, Choi JY, Seo BF, Moon SH, Oh DY, Ahn ST, Rhie JW (2014). Treatment for intramuscular lipoma frequently confused with sarcoma: a 6-year restrospective study and literature review. Biomed Res Int.

